# Blue velvet – Pseudocyanosis, a rare case of drug-induced bluish hyperpigmentation with clozapine: A case report

**DOI:** 10.1097/MD.0000000000044120

**Published:** 2025-09-05

**Authors:** Tina Mazza, Antoine David, Gürkan Kaya, Sébastien Menzinger, Amandine Berner, Jacques Serratrice, Matteo Coen

**Affiliations:** a Division of General Internal Medicine, Department of Medicine, University Hospitals, Genève, Switzerland; b Department of Dermatology and Venereology, University Hospital of Geneva, Genève, Switzerland; c Department of Clinical Pathology, University Hospital of Geneva, Genève, Switzerland; d Unit of Development and Research in Medical Education, Faculty of Medicine, University of Geneva, Genève, Switzerland.

**Keywords:** atypical antipsychotics, case report, pigmentation, skin discoloration

## Abstract

**Rationale:**

This case highlights the importance of considering a wide range of possible diagnoses when faced with unexplained hemorrhagic symptoms. When standard investigations fail to identify a clear cause, it is essential to conduct a detailed dietary history. This can lead to the diagnosis of scurvy, a reversible vitamin C deficiency that is often overlooked in populations at risk.

**Patient concerns:**

The patient is a 55-year-old man with well-controlled Crohn disease who presented with unexplained bilateral leg pain and extensive hematomas on his lower limbs, significantly affecting his mobility and raising concerns about a serious condition.

**Diagnoses:**

The final diagnosis was scurvy, resulting from a chronic deficiency of vitamin C due to a severely limited diet that lacked fruits and vegetables. Initially, differential diagnoses included deep vein thrombosis and drug-induced coagulopathy. However, a physical examination revealed follicular purpura, and the identified dietary restrictions ultimately led us to the correct diagnosis and enabled effective treatment.

**Interventions:**

Vitamin C supplementation was initiated.

**Outcomes:**

Timely identification and vitamin C supplementation resulted in a rapid improvement in the patient’s condition.

**Lessons:**

This case highlights the importance of recognizing nutritional deficiencies in modern medicine, especially for patients with chronic illnesses. It demonstrates that classic diseases, such as scurvy, can still occur and be overlooked if comprehensive patient histories and thorough examinations are not conducted.

## 1. Introduction

Cyanosis is a bluish or grayish discoloration of the skin or mucous membranes, indicating hypoxemia and requiring prompt evaluation.^[1]^ However, not all bluish skin indicates hypoxia. Pseudocyanosis mimics cyanosis without arterial oxygen desaturation, often due to drug use or heavy metal exposure, which can lead to diagnostic confusion.^[2]^ While classical antipsychotics like chlorpromazine are associated with cutaneous hyperpigmentation, evidence for atypical antipsychotics is limited. This report describes a rare case of drug-induced pseudocyanosis in a patient who has been using clozapine and risperidone long-term, highlighting the need to distinguish between true cyanosis and pseudocyanosis and the importance of histological evaluation.

## 2. Case presentation

**Figure 1.**
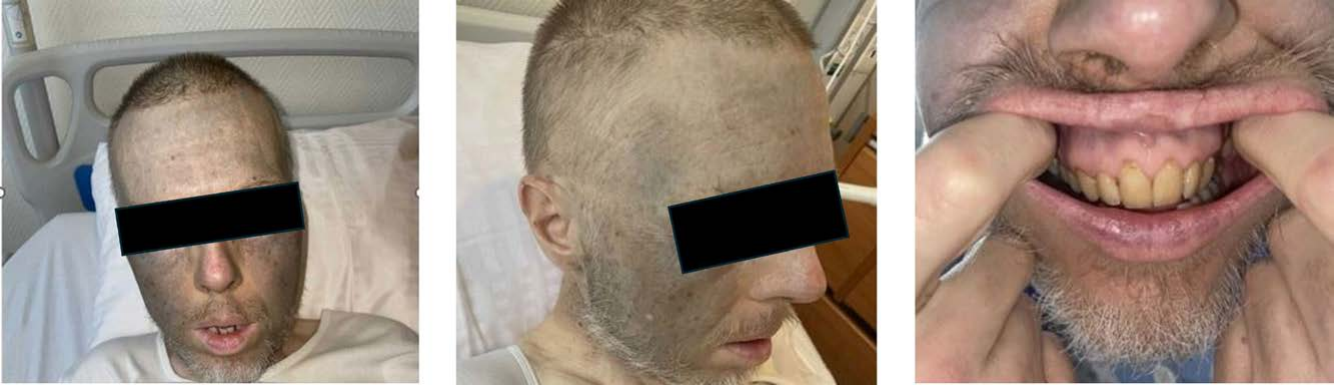
Cyanosis of the face respecting the nose and the perioral area, slightly involving the gums.

**Figure 2.**
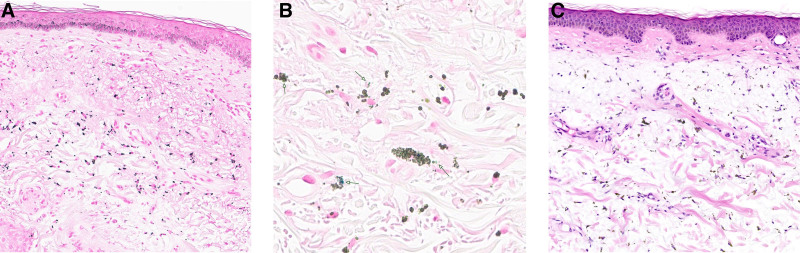
Histological staining of skin sections. The epidermis appears atrophic in all samples. The dermis exhibits prominent actinic elastosis, as shown by hematoxylin and eosin staining (A, 20×) and Masson trichrome staining (C, 20×). Brown pigment deposits are also present, as indicated by the arrow in the Persian blue-stained section (B, 60×).

A 59-year-old male patient was admitted to our department due to the onset of acute febrile confusion that developed over the previous 24-hour period. Clinical examination revealed isolated cyanosis of the face, neck, and nape, which persisted under pressure (Fig. 1). Cardiopulmonary examination was found to be normal. Initial blood gas measurements indicated a pH of 7.41, a PaCO_2_ of 40 mm Hg, a PaO_2_ of 90 mm Hg, a SaO_2_ of 98%, and a HCO_3_^−^ of 25 mEq/L, along with a methemoglobinemia level of 0.7%. A chest X-ray indicated clear lung fields with no signs of infiltrates or opacities. Initial laboratory data revealed a hemoglobin level of 114 g/L, a white blood cell count of 4.6 × 10^9^/L, and a platelet count of 203 × 10^9^/L. Liver function tests showed aspartate aminotransferase at 310 UI/L (normal range: 14–50) and alanine aminotransferase at 114 UI/L (normal range: 12–50). A nasopharyngeal swab analyzed via polymerase chain reaction confirmed the presence of influenza A infection. Despite rapid clinical improvement with the administration of oseltamivir, the bluish skin pigmentation remained unchanged. A skin biopsy disclosed brownish pigment deposits within macrophages, which tested strongly positive for Masson-Fontana staining, indicating drug-induced hyperpigmentation (Fig. 2). Given the persistent bluish appearance of the face and the absence of hypoxemia, a diagnosis of pseudocyanosis was considered. The differential diagnosis for pseudocyanosis includes heavy metal poisoning due to exposure to silver, gold, or mercury; hemosiderosis; exogenous or endogenous ochronosis, including that caused by alkaptonuria; specific dermatological conditions such as pigmented lichen planus; and drug-induced pigmentation. In the case of our patient, no reported history of exposure to silver or mercury was evident. He reported oral iron supplementation for iron absorption issues since 2017, and his ferritin level was measured at 82 µg/L (normal range: 11–342 µg/L). Furthermore, the skin biopsy exhibited no evidence of iron deposition, effectively ruling out other differential diagnoses, including argyria and chrysiasis. The patient reported that he had experienced this discoloration for over 20 years, which coincided with a chronic psychotic disorder. He had been treated with clozapine at a dosage of 400 mg/day and risperidone at a dosage of 2 mg/day since 1998. Notably, he had never received chlorpromazine. Olanzapine was initially prescribed at the time of his schizophrenia diagnosis but was discontinued after 1 month due to the lack of efficacy. Additionally, he reported oral iron supplementation as previously mentioned, along with bisoprolol for managing cardiac arrhythmias induced by clozapine since 2012. Given the stability of his mental health and the absence of any symptoms related to the pigmentation, the patient expressed no interest in altering his current treatment regimen.

## 3. Discussion

Cyanosis is characterized by a blue or grayish discoloration of the skin or mucous membranes due to increased levels of reduced circulating hemoglobin, which can result from various causes.^[1]^ Several drugs and heavy metals can induce a bluish or slate-gray pigmentation of the skin, which may be mistaken for cyanosis. This type of skin discoloration is known as pseudocyanosis and must be distinguished from true cyanosis.^[2]^ The pathophysiology behind this condition involves various mechanisms, including melanin accumulation, lipofuscin synthesis, or the direct accumulation of the drug itself, resulting in extracellular granules or foreign bodies that macrophages cannot eliminate, as seen in our case. Hyperpigmentation due to antipsychotics has been documented since 1964,^[3]^ with chlorpromazine being the first and most widely used conventional antipsychotic associated with skin pigmentation resembling cyanosis. More recent atypical antipsychotics have also been implicated in cutaneous hyperpigmentation.^[4]^ In our case, the patient underwent long-term treatment with clozapine and risperidone. Although there have been no reported cases of bluish hyperpigmentation due to clozapine, Borovik et al described a 55-year-old patient on long-term clozapine who developed brownish ocular and cutaneous hyperpigmentation in sun-exposed areas.^[5]^ Regarding risperidone, 2 cases of hyperpigmentation have been noted: one by Bains et al involving a 22-year-old patient with generalized blue hyperpigmentation after 1 month of treatment^[6]^ and another by Saha et al describing reticular palmar hyperpigmentation after 7 months of treatment.^[7]^ While clozapine is not typically associated with bluish hyperpigmentation, its partial agonistic action at dopamine D2 receptors may influence melanocyte activity, leading to pigmentation changes. Dopamine receptors are involved in melanogenesis, and clozapine interaction with these receptors could contribute to pigmentation alterations in the skin.^[8]^ This case emphasizes the importance of recognizing drug-induced hyperpigmentation, including its rare presentation with atypical antipsychotics. It highlights the need for clinicians to consider this when evaluating patients undergoing long-term antipsychotic therapy.

## 4. Conclusion

Clinicians should be aware of drug-induced pseudocyanosis. When presented with a bluish skin appearance, normal PaO_2_ levels, and normal hemoglobin concentrations, one should consider the possibility of abnormal hemoglobin or abnormal skin pigmentation. Pseudocyanosis can usually be distinguished, as it does not blanch under pressure.

## Author contributions

**Conceptualization:** Gürkan Kaya, Sébastien Menzinger, Amandine Berner, Jacques Serratrice, Matteo Coen.

**Data curation:** Gürkan Kaya, Jacques Serratrice.

**Investigation:** Antoine David.

**Supervision:** Matteo Coen.

**Validation:** Amandine Berner, Matteo Coen.

**Writing – original draft:** Tina Mazza, Antoine David, Jacques Serratrice, Matteo Coen.

**Writing – review & editing:** Tina Mazza, Sébastien Menzinger, Amandine Berner, Jacques Serratrice, Matteo Coen.
